# Villains or victims? An ethnography of Afghan maternity staff and the challenge of high quality respectful care

**DOI:** 10.1186/s12884-019-2420-6

**Published:** 2019-08-22

**Authors:** Rachel Arnold, Edwin van Teijlingen, Kath Ryan, Immy Holloway

**Affiliations:** 1 Bournemouth University, Faculty of Health & Social Sciences, Bournemouth House, 19 Christchurch Road, Bournemouth, BH1 3LH UK; 2 Bournemouth University, Centre for Midwifery, Maternal & Perinatal Health, Bournemouth House, 19 Christchurch Road, Bournemouth, BH1 3LH UK; 3 University of Reading, School of Pharmacy, Whiteknights, Reading, RG6 6AH UK; 4 Bournemouth University, Centre for Qualitative Research, 19 Christchurch Road, Bournemouth, BH1 3LH UK

**Keywords:** Afghanistan, Ethnography, Midwifery, Facility-based childbirth, Mistreatment, Respectful care, Quality of healthcare, Health personnel, Low and middle income countries

## Abstract

**Background:**

Healthcare providers are the vital link between evidence-based policies and women receiving high quality maternity care. Explanations for suboptimal care often include poor working conditions for staff and a lack of essential supplies. Other explanations suggest that doctors, midwives and care assistants might lack essential skills or be unaware of the rights of the women for whom they care. This ethnography examined the everyday lives of maternal healthcare providers working in a tertiary maternity hospital in Kabul, Afghanistan between 2010 and 2012. The aim was to understand their notions of care, varying levels of commitment, and the obstacles and dilemmas that affected standards.

**Methods:**

The culture of care was explored through six weeks of observation, 41 background interviews, 23 semi-structured interviews with doctors, midwives and care assistants. Focus groups were held with two diverse groups of women in community settings to understand their experiences and desires regarding care in maternity hospitals. Data were analysed thematically.

**Results:**

Women related many instances of neglect, verbal abuse and demands for bribes from staff. Doctors and midwives concurred that they did not provide care as they had been taught and blamed the workload, lack of a shift system, insufficient supplies and inadequate support from management. Closer inspection revealed a complex reality where care was impeded by low levels of supplies and medicines but theft reduced them further; where staff were unfairly blamed by management but others flouted rules with impunity; and where motivated staff tried hard to work well but, when overwhelmed with the workload, admitted that they lost patience and shouted at women in childbirth. In addition there were extreme examples of both abusive and vulnerable staff.

**Conclusions:**

Providing respectful quality maternity care for women in Afghanistan requires multifaceted initiatives because the factors leading to suboptimal care or mistreatment are complex and interrelated. Standards need enforcing and abusive practices confronting to provide a supportive, facilitating environment for both staff and childbearing women. Polarized perspectives such as ‘villain’ or ‘victim’ are unhelpful as they exclude the complex realities of human behaviour and consequently limit the scope of problem solving.

## Background

More often than not, debates on the quality of maternity care primarily focus on healthcare providers. Globally, their attitudes, behaviours and reputations influence women’s choices regarding ante-natal attendance, place of birth and the use of biomedical or traditional health services [1]. The efficacy of facility-based maternity care is also directly linked to the competencies and motivation of healthcare providers [2, 3].

Many studies have highlighted that health systems have failed women during pregnancy and childbirth [4]. In low, middle and high-income countries their wishes have been ignored, they have received poor quality care, been humiliated, neglected, ridiculed, discriminated against and physically abused [5–8]. Prioritising quality, preventing unnecessary deaths from suboptimal care [2, 9] and ensuring women in facility-based childbirth are treated with kindness and respect requires an in-depth understanding of the current deficits. Crucially, it requires strategies that ensure maternity healthcare providers work with diligence, professionalism and kindness. Few studies, however, have sought to understand the attitudes and behaviours of midwives, doctors and care assistants [10].

The paradox is that despite professional training many health providers do not provide good quality care [2]. Furthermore, some do not treat women in childbirth with respect and kindness, be it out of ignorance, restrictions at work or in society or alternatively simply ill will [11, 12]. One explanation centres on tough working conditions for staff that might contribute to poor quality care [10]. Another is that violence in the surrounding environment and the lack of status for women in society might drive mistreatment [13, 14]. Yet another explanation centres on healthcare provider ignorance or disregard for the rights of childbearing women and suggests the need for training, behaviour change strategies and accountability mechanisms [15–17]. Understanding and addressing the barriers to high quality healthcare requires careful investigation, “*going deep into the messy realities of health services, to understand local problems, find innovative solutions, learn from mistakes, scale up what works, share experiences”* [18].

For more than four decades Afghans have suffered political upheaval, violence, migration and conflict [19]. The hoped for peace following the fall of the Taliban regime (2001) has not materialised. Even in Kabul city, despite a semblance of normality, there is the constant threat of unpredictable violent attacks for all - including healthcare providers travelling to work. The health system infrastructure was all but destroyed during the height of the conflict and many professionals fled the country [20, 21]. Despite the immense national and international efforts to rebuild the health system [22, 23], difficulties in assessing care due to insecurity, poverty or terrain, as well as suboptimal care and political interference continue to undermine its effectiveness [24, 25].

Improving women’s health was one of the top priorities of the post-Taliban Afghan Ministry of Public Health (MoPH), international partners and donors in 2002 as the country faced a maternal mortality ratio that was, in one district, unenviably the highest ever recorded globally [26]. Attention was particularly focused on increasing the number of female health workers able to provide skilled birth attendance [27]. Midwifery schools were opened in each province using a new standardised evidence-based curriculum and competency-based learning approaches [28]. Since 2003 over 4,600 midwives have been educated [29] and newly graduated female doctors have resumed residency training in obstetrics and gynaecology. The proportion of births attended by skilled providers has risen from less than 10% in 2003 to 51% in 2015 [27, 30]. Reviews of clinical decision-making, maternal and perinatal outcomes, however, raised concerns regarding the quality of care in Afghan public health facilities [31, 32]. In addition, out of pocket expenditures deter women from accessing care [33] and women complain that maternity health professionals are unfriendly, use harsh words and even physical violence [34]. Recent surveys estimate that the maternal mortality ratio remains one of the highest globally at 1,291 deaths per 100,000 live births [30, 35]. Perinatal mortality rates (which are comprised of stillbirths and neonatal deaths within the first seven days of life) are estimated at 36 deaths per 1,000 pregnancies although the report noted that neonatal deaths appeared to be under-reported [30]. These figures highlight that increasing healthcare provider numbers is not enough; there must be a corresponding increase in the quality of care to achieve the best outcomes for mothers and their newborns. This qualitative research in a Kabul maternity hospital explored the experiences, perspectives and motivations of the doctors, midwives and care assistants. It studied the day-to-day realities of Afghan maternity staff and the roots of poor quality care in relation to their roles and responsibilities. The behaviours of healthcare providers and their contributions to respectful care, mistreatment or poor outcomes were examined in light of a narrative that oscillates between two positions: healthcare providers as the villains or as the victims.

## Methods

This ethnographic study [36] was conducted by the first author in two phases in a busy tertiary maternity hospital in the Afghan capital Kabul between 2010 and 2012. It commenced with over six weeks of participant observation in all areas of the hospital during mornings, afternoons, evenings and nights [37]. Informal group discussions and numerous conversations occurred spontaneously with staff as they explained the systems, pressures, joys and frustrations of their work. Field notes and memos were taken during observation. Semi-structured interviews [38] were then conducted with 23 hospital staff members who were asked about care in the hospital, their roles, and ideas. A broad range of staff was interviewed including senior and junior midwives, obstetricians and gynaecologists, resident doctors and care assistants. (The care assistants, or *khālas,* were untrained female workers who transferred women between wards, washed and dressed newborns, ran errands for staff and women in childbirth, conveyed messages to relatives, cleaned and controlled entry to the obstetric wards). The selection of participants was a mixture of purposive sampling, opportunistic and self-selection to ensure that a wide range of views was represented. Staff who appeared particularly informative or knowledgeable were asked if they would give us an interview. In addition, we announced at staff meetings that anyone was welcome to talk to us - four resident doctors and one senior midwife volunteered for an interview. Ongoing informed consent was obtained from all participants. An aide memoire was used as an interview guide but after the initial questions the interview was adapted to the participant and explored issues that they considered important. Some participants needed few questions or prompting, others more. Interview questions evolved during data collection as new understandings and perspectives of what was important to healthcare providers developed. This flexibility gave the opportunity to hear unexpected perspectives and not be limited by initial assumptions. Interviews lasted 20–90 min and were digitally recorded with permission then transcribed by the first author. Alternatively, handwritten notes were taken during the interview then later checked with the interpreter to ensure important information had been recorded (Table 1).

**Table 1:** Overview of study participants

Type of interview	Participants
Background interviews	19 Afghans
	22 non-Afghans
Semi-structured interviews	1 hospital manager
	5 obstetrician/gynaecologists
	6 resident doctors (year 1–4 of residency programme)
	2 senior midwives
	8 midwives (6 months–10 years’ experience)
	1 care assistant
Informal group discussions	Group of obstetrician/gynaecologists
	Group of resident doctors
	Group of midwives and nurses
	Group of care assistants
Community focus group discussions	FGD1, 6 female members of an extended family
	FGD2, 10 women from very poor area of Kabul

Focus group discussions (FGDs) were conducted during this time with two groups of women from different Kabul communities to understand their experiences and priorities regarding care [39]. One FGD was held in the home of a community leader with six female members of his extended family. The second FGD was held in a poor area of Kabul with ten women who were members of a pre-existing self-help group. The main purpose of the FGDs was to understand what was important to Afghan women when they were in childbirth and to interpret maternity care from their perspective rather than from Western notions of care. For this reason it was decided that any women were eligible for the FGD regardless of where they had delivered their baby. Verbal consent was given by both groups for their discussions to be digitally recorded. The first FGD lasted 93 min and the second 45 min.

Forty-one background or key informant interviews were undertaken with Afghans (*n* = 19) and non-Afghans (*n* = 22). Informants were selected for their expert knowledge and were either already known to the first author, were met during the course of the study or introduced as someone who could provide an important perspective. These interviewees had in-depth knowledge of pertinent issues including conducting research in Afghanistan, the health system, the MoPH, midwifery and medical education, the culture, linguistics, education, and the impact of recent history on society and mental health. Background interviews were conducted at the commencement of the study and throughout. They were a safeguard against forming premature judgements and provided broad perspectives on the Afghan health system and the society that defines the healthcare providers.

As a cross-cultural study that depended on translation, we took care to ensure the quality of translation throughout. Prior to the study commencement two Afghan researchers advised on the correct translation of key concepts and words. Information sheets and consent forms were translated into Dari and Pashtu, the two main Afghan languages. These were then ‘back translated’ into English by different Afghan translators to ensure accuracy [40]. A female Afghan interpreter was recruited locally and trained specifically for this research. She accompanied the first author and interpreted informal conversations throughout participant observation, staff meetings and semi-structured interviews [41]. An Afghan midwife researcher later transcribed and translated digitally recorded interviews as a quality control measure. Ethical approval for this study was given by the Afghan MoPH Institutional Review Board and by Bournemouth University, UK.

Thematic analysis was used to analyse the data manually [42]. Data was coded section-by-section often using ‘in vivo’ codes or labels that came from the words or phrases of the participants. Similar codes were grouped together into categories and then combined into more conceptual themes [43]. Initially FGDs, field notes from observation and background interviews were analysed individually. Semi-structured interviews were also analysed separately by professional grouping and level of seniority such as junior and senior midwives, resident doctors and senior doctors. Finally, a broad framework was developed to combine, compare and refine categories from all the different types of data. The first author did the majority of the analysis and ET and KR analysed some interviews. The developing and final themes were discussed and agreed with all authors.

The thematic analysis resulted in five themes: the culture of care; challenges of care; motivation; family and social influences; fear, power and vulnerability. This paper focuses on the themes ‘culture of care’, ‘challenges of care’ and ‘fear, power and vulnerability’ but incudes links to the other two themes as they are intertwined.

## Results

The findings first explore the notion that healthcare providers are villains, secondly that they are victims, and finally present a more nuanced understanding of the role of the healthcare providers in suboptimal care.

### Healthcare providers as villains

Women in the FGDs had delivered their babies in various maternity hospitals but were generally dissatisfied with their care. Once they had been admitted, they explained, they received little attention or monitoring. “*No one feels responsible*” one woman asserted. Many women said no one explained anything to them and they had given birth to their babies alone as this woman’s experience illustrates:

I delivered on the floor of the corridor, I didn’t understand anything, people were watching and finally a patient called the doctor and a cleaner to come and take care of me. (Community FGD2)

A midwife criticised her colleagues for the lack of postnatal checks:

After women deliver they [=midwives] *have* to check their blood pressure, their bleeding, everything – but they are not checking these things. When women deliver at night, in the morning they say…‘*go home!’* [Without first doing their observations] This is the care of this hospital. (Experienced midwife)

The women said that they were made to pay: for medicines that should be free, to ‘speed up’ the labour, to receive a blanket, have the heater put on and to celebrate the arrival of their baby (more for a boy, less for a girl). The women were convinced that operations were performed unnecessarily to gain money or for practice. Women also had to recompense staff for the time they spent looking after them. “*Money changes behaviour”* a woman in the FGD explained. When paid, “*angry staff become very kind*”. It was not enough to give “*some money*”, however, it had to be “*enough money*”. Several interviewees explained that once staff realised a woman in childbirth was wealthy they demanded more money from her relatives. The cost of having a baby in a health facility is prohibitive for the poor, a female doctor confirmed:

In Kabul there are many issues that deter women from delivering in hospital but the majority of women don't go to hospital because of poverty. (Background interview - female doctor working in the community)

Although women in the FGDs were from different social, ethnic and educational backgrounds, being treated with politeness, kindness and respect was vitally important for them all. “*Patients are very sensitive*” several women explained. They felt, however, that the staff did not care but were “*busy with other things, joking, chatting, telling stories not paying attention to the patients*”.

Doctors tell us they are tired of lots of births, they say ‘*Please stop getting pregnant*’…they use bad words to us and slap us – they say *‘When you had sex with your husband you should have thought about the pain you have now’*. (Community FGD 2)

During observation women were often left alone until the baby’s birth was imminent. Women were not permitted to have relatives with them during labour due to the lack of space. Young women having their first babies were particularly distressed but their cries for help, for their mothers or for God were largely ignored. Doctors and midwives explained that some women control themselves and some don’t. A sense of vulnerability and fear pervaded women’s accounts of care in Kabul’s maternity hospitals as illustrated by this woman’s recollections:

When I saw the behaviour of doctors I got scared and thought they are getting women ready for the butcher, for slaughtering. (Community FGD2)

This analogy, likening women in childbirth to helpless animals about to be killed by a butcher, is particularly shocking in a culture where it is offensive to liken someone to an animal. A female Afghan doctor was also shocked by how frightened women were of the healthcare providers. She recounted a discussion in the MoPH regarding a woman in a Kabul hospital: unnoticed by staff the woman had suffered a postpartum haemorrhage and was discovered dead in her bed:

You know it was really shocking for me to realise how much the mothers are afraid of the hospital staff, afraid to say *‘help me, something is wrong’.* (Background interview - female doctor working in the community)

The accounts of women who had given birth in Kabul hospitals, background interviewees, personal observations and healthcare providers themselves concurred that although some staff provided appropriate care and were kind, substandard care and mistreatment did occur in Kabul maternity hospitals.

### Healthcare providers as victims

Interviews and informal conversations with healthcare providers revealed a challenging, punitive and stressful working environment. Many interrelated issues affected the ability and motivation of staff to provide respectful high quality care.

One 60-bedded area of the hospital included labouring and postnatal women, high risk and intensive care rooms, with women often sharing beds. During the night two resident doctors and three midwives covered this area, often there was only one doctor and two midwives.

If we had less patients, a midwife explained, then we could care but with so many we can’t. This is our hope to care for patients better. We know how to care, like we were taught in the school, but we can't do it here. (Field notes day 4)

Another midwife illustrated their predicament:

When the doctors and midwives are caring for one patient another patient delivers on the ground. (Hospital midwife)

A hospital team was endeavouring to ensure that the partograph was used for every woman but several staff asserted that this was not possible. A resident doctor explained the conflict between the policy and her workload:

We have the partograph and learn how to use it, but how can we manage when we have 35 patients in delivery during the night? Oh, it’s so difficult, really difficult! (4^th^ year resident doctor A)

The high numbers of women in normal childbirth deprived severely ill women from the care they needed; the ambulant women were also more able to demand attention. Comparing the numbers of staff on duty with potential numbers of women in childbirth, including women with complications, it was reasonable to conclude that high quality care for all was impossible. Individual staff members were nonetheless blamed if things went wrong.

Many doctors, midwives, care assistants and senior hospital staff complained of chronic illnesses. They attributed this to the pressure and stress of the workload, long duties and the lack of a shift system. When they became tired, they explained, they made mistakes and lost patience with the women they were caring for. A resident doctor admitted that her energy and kindness sometimes ran out:

We should be kind with the patient, we are not kind enough because there are too many patients… If I care for 10 or 15 patients, after that I have no energy so when they ask something I say ‘*Be quiet! I have no time!’* In our heart we know that we have done things wrong… but the pressure of many patients makes it so very difficult. (4^th^ year resident doctor B)

When asked the reasons that care was at times suboptimal, another resident doctor exclaimed:

Tiredness! When people are tired they become crazy, they fight and shout and make mistakes! I also get tired, tired of many patients and switch off to their cries (4^th^ year resident doctor A)

Doctors and midwives were powerless to control the workload as the hospital director forbade them from turning away any labouring woman. Although it was women in childbirth who primarily suffered as a result of inadequate staffing levels and staff exhaustion, doctors and midwives said they lived in fear of making mistakes, and being held accountable.

Many healthcare providers felt unappreciated, under-valued and unsupported by management. Those who worked well, they complained, were treated the same as those who did not. “*We have this pain in our hearts*” a midwife explained, pain at being criticised, not appreciated or listened to. Another midwife explained how management dealt with complaints about staff:

If a midwife makes a mistake the hospital directorate immediately sends a warning letter. Instead of sending warning letters they can invite the midwife to the office, talk to her and advise her… When a patient complains they do not ask us ‘*is this complaint true, did you commit this mistake*’? (Experienced midwife)

“*No-one listens to our ideas – so we are silent because of that*”, a midwife said. Other staff said that if they complained they were told they could leave. A newly qualified midwife concurred:

Senior managers say ‘*if you have the ability stay, if you cannot tolerate it,* [the workload and conditions implied] *then leave. There are many jobless midwives and doctors, I can fill your place’*. (Newly qualified midwife)

Although there were exceptions, the stories of staff portrayed a lack of teamwork and kindness between colleagues. One healthcare provider, the only wage earner in her extended family, had chronic and debilitating health problems:

When I explain my health problem to other staff they say ‘*What should I do about it? It’s not my problem’*. No one is kind or helps me, they shout at me and say they will replace me if I don't do my work. (Healthcare provider)

The lack of collegiality was compounded by the practice of quickly apportioning blame every time an adverse incident occurred. A newly qualified specialist doctor explained that blaming others was a survival strategy.

Mistakes happen and from the beginning doctors learn to defend themselves: one-way is to blame others. (Newly qualified obstetrician/gynaecologist)

Dysfunctional relationships between staff members undermined communication and consequently clinical care. A resident doctor explained that one day she commenced her duty unaware that there was a seriously ill eclamptic woman in another ward. She spent the first hour examining women in labour. By the time she reached the eclamptic woman it was too late: the woman died shortly afterwards.

The acquisition and use of clinical skills by doctors and midwives are vital if women are to receive quality care. In this hospital, however, learning and utilising clinical skills was a complex issue. Although there was a training programme, residents explained, those without connections to a trainer or senior doctor had no option but to teach themselves clinical skills.

So day and night we work by ourselves and teach ourselves. For example, if the first time I do something I feel it was not correct, then the next time I change it. I am a fourth year resident, but I don't feel that I learnt from anyone, nobody took my hand and showed me ‘*do it like this, don't do it like that’*. (4^th^ year resident doctor B)

However diligent they were, resident doctors without connections were seriously disadvantaged and more at risk of making clinical errors than their colleagues. If a woman or her baby suffered serious complications or died maternity staff could be physically assaulted by the family or taken to court. The absence of professional indemnity insurance increased the pressure on them. “*We are stressed every day”*, a resident explained, worried that they would make a mistake. The lack of in-service training opportunities also resulted in profound frustration as illustrated by a resident doctor:

Many times we contact the training centre and say: ‘Train us because we don't know what we are doing, we are like wild people, like wild animals, we do whatever we want with the patients, please train us.’ They told me, ‘*Your turn will come*’. I don't know when. (2nd year resident doctor)

The midwifery education programme equipped midwives with the skills to manage most obstetric complications. Once graduated, however, new midwives said they were only allowed to help women having their second or subsequent baby. Experienced midwives were also frustrated at their limited scope of practice; they said they felt sad and regretted choosing midwifery as their profession.

We would like to put everything we have studied into practice. Because this does not happen midwives lose interest; this is the reason some midwives want to leave this hospital. (Experienced midwife)

The midwives’ scope of practice was not formally assessed but generally doctors admitted and assessed women in labour while midwives took blood pressures, gave injections, commenced intravenous infusions and made beds. In the delivery area doctors often sutured episiotomies on multiple women and managed some deliveries. The resistance from doctors and some senior midwives to the extended midwifery role wasted resources, put unnecessary pressure on resident doctors and, most importantly, increased the likelihood of avoidable mortality and morbidity for mothers and their unborn babies.

Finally, the healthcare providers were not passive protagonists in poor quality care. Some healthcare providers felt deeply about the unnecessary suffering of women in childbirth and the lack of high quality care.

When I see the patients in here I want to cry. We cannot put our sisters in such a place. (4^th^ year resident doctor C)

These healthcare providers were active and bold in trying to improve standards, they were enthusiastic about quality, teaching and encouraging colleagues.

### The role of healthcare providers more nuanced than villain/victim

Underlying notions of healthcare providers as either villains or victims were revealed during data collection. One foreigner, a healthcare professional who was struggling to improve standards of care deliberated whether “*carrot or stick*” approaches would be the most effective strategy. Our findings, however, revealed the inadequacies and dangers of such binary thinking.

In many ways the staff were victims of a system that did not facilitate their ongoing learning, provide the support structures, enabling environment or clinical resources that they needed to care. The role of healthcare providers in suboptimal care, however, was complex. For example, among the staff who complained that they were treated poorly and unfairly by management were staff whom women in the community accused of stealing the medicines that they sent to their relatives in labour. Many healthcare providers complained that care suffered through a lack of supplies such as gloves, medicines and equipment. The lack of essential supplies was not simply a procurement issue or high patient numbers but as one resident explained – “*there are fights over supplies*”. Not enough supplies were provided she claimed, and then some were taken by individual staff to be used in their private clinics or sold in private pharmacies.

Six weeks of observation confirmed that this was an extremely busy hospital. The majority of staff claimed that the excessive workload and the high number of women with complications was the reason they could not care as they had been taught. If patient numbers were limited, staff asserted, standards of care would improve. Several midwives and doctors reflected, however, that there were other reasons for poor care and the lack of monitoring.

They use patients as an excuse – sometimes they are not busy but still don't work well. (Experienced midwife)

The taking of bribes could be linked to the poor economic situation of staff, the low salaries and high levels of unemployment in Kabul that meant some staff were the sole wage earner for their extended family. While it could be argued that economic necessity was the reason that staff asked for or demanded money, several interviewees suggested that even if salaries were increased they were not sure that this practice would stop.

Some of them are very poor, they take money to pay the rent or for their children’s education but some of them are very rich and taking money is just their habit. (Obstetrician/gynaecologist)

Although midwives and doctors struggled to acquire or to use their skills, once acquired, skills could be used for additional income in the private clinics of some doctors and midwives. A woman in the community FGD complained that healthcare providers were “*playing with the lives of people*”, telling women lies about their condition, leading to unnecessary treatments or surgery to earn extra money. One midwife demands money for each suture, another woman explained. She tells her clients “*if you are paying me I will continue suturing, if not I will leave it as it is”.*

Study findings revealed that poor quality disrespectful care occurred not only because of practical issues such as the workload, staff exhaustion, shortage of supplies or a lack of skills but also because the institutional culture was not conducive to kindness or respect. Powerful staff were working against positive change an experienced midwife explained:

New midwives want to change but when faced with the senior midwives they can’t do it. If they start to treat patients nicely and not shout they get a lot of abuse. If they don't behave in the accepted way they face a hard time and can be sent to the laundry [to work]. (Background interview - experienced Afghan midwife working for NGO)

Punishment in this instance was not given for poor care or negligence of women in childbirth; punishment was given to staff who were kind thereby challenging the status quo. It was hard for individuals to stand up against the behavioural norms and senior staff.

Doctors and midwives complained that management did not care. Rather than check on women in childbirth, they explained, management visits to the clinical areas tended to focus on unimportant issues such as confiscating water-heating elements to save electricity. Furthermore, staff trying to improve the quality of care felt unsupported as their requests to management for letters appreciating staff members who were working well had been ignored.

## Discussion

Providing high quality respectful care for women in childbirth and preventing unnecessary mortality and morbidity for them and their unborn babies requires a transformation of the health workforce [9]. Finding effective strategies to improve health worker performance, however, has been challenging [44]. Helping the workforce be motivated and effective, or *“optimising the workforce”* ( [2], p2311) requires a thorough understanding of them so that strategies can be based on their precise situation and needs, rather than assumptions. General overviews provide broad brush-stroke insights [10, 13]. This paper, however, provides an in-depth analysis of a mixed group of Afghan maternity care providers in a real-life setting, a low-income fragile state [45]. It highlights their dilemmas, different levels of commitment, notions of being able to make a difference, and the obstacles preventing them from providing better care.

In some studies, the reasons for health worker behaviours and ideas for addressing them have been based on the ideas of women they have cared for or on literature from other contexts rather than asking doctors and midwives themselves [16]. Bradley and colleagues [46], pointed out that midwives’ voices have been largely missing from the discourse. They based their conceptual framework of healthcare provider behaviours, however, on the assumptions of women they had cared for [46]. We would dispute the premise that a “*robust understanding of the factors driving disrespectful care”* can be developed from conjecture ([46], p166). Our findings demonstrate the complexity of institutional cultures and the dangers of making judgements without gaining first-hand insights from healthcare providers themselves.

### Danger of the villain approach

Women’s experiences of suboptimal care and mistreatment in facility-based childbirth are unacceptable [47]. An underlying notion of healthcare providers as predominantly ‘to blame’ for poor standards of care and mistreatment, however, is also unacceptable. The combination of heavy workloads and inadequate numbers of health workers leading to staff burnout and poor interactions with women in childbirth is frequently documented in low and middle-income countries (LMICs) [48–50]. Six weeks of observation confirmed the overwhelming demands staff faced working a night shift, for example, in this overcrowded, under resourced tertiary maternity hospital. On many occasions it was humanly impossible to provide care for all, much less anything resembling quality care. As Freedman and Kruk [51] argued, disrespect and abuse are inflicted, not just by individual providers, but by health systems as a whole when the lack of personnel, vital infrastructure and supplies make care impossible. Our findings concur, that judging healthcare providers on generic standards written far from the messy reality of health facilities in LMICs, is grossly unfair because it demands the impossible. In addition, if educating staff about childbearing women’s rights to respectful care becomes the major intervention [15, 16] the underlying narrative implies that addressing staff ignorance will prevent mistreatment.

Furthermore, strategies that solely focus on improving healthcare provider standards of care and behaviour assume high levels of individual agency. The evaluation of a multicomponent behaviour change intervention in Kenya concluded that although positively influencing providers understanding of client rights is feasible providers were unable to apply this to the workplace because of peer influences [49]. In a Palestinian study [48] maternity care providers felt trapped and hopeless in a health system where poor standards of care were the norm, where no one listened to them, or cared. The Palestinian nurses and midwives had no means to advocate for change and if they used their skills to help women, for example, by suturing episiotomies, they were punished. Social norms and pressures limit the individual agency of doctors, midwives and care assistants in many LMICs [52–54]. Similarly, Afghan midwives complained that no one listened to their ideas. If they spoke out about working conditions they were told they could resign, if they challenged the institutional culture by, for example, ‘treating women kindly’, they were threatened or punished.

We would argue that approaches that frame midwives, doctors and care assistants as ‘villains’ demotivates and alienates those who are trying hard to ensure that women give birth safely. It also prevents outsiders from listening to and learning from their experiences, perspectives and suggestions. Most crucially, the focus on service providers deflects attention and blame from structural weaknesses in the health system. More promising approaches are multicomponent interventions that include analysis of the broader health system, supportive rather than punitive supervision for staff that listens to their voices, and respects their rights alongside those of the women for whom they care [55, 56].

### Danger of the victim approach

Many studies especially from LMICs portray the challenging work environments midwives, doctors, nurses and care assistants experience [50, 57]. It is acknowledged that low salaries, unsafe working conditions, lack of essential supplies, high workloads, low status of women and violence in society contribute to poor standards of care, staff burn out and the mistreatment of women in childbirth [13, 58]. Staff members in our study had legitimate grievances such as the lack of essential supplies or difficulties gaining or using their skills and an excessive workload. To focus on those grievances, however, without exploring other factors can tend towards excusing the inexcusable and abrogating personal accountability. The danger of the ‘victim’ narrative is that it tends to absolve healthcare providers en masse for their actions and omissions. There needs to be an awareness of the potential of some staff not only for unintended mistakes but also for deliberate neglect, cruelty or extortion. As Afghan MoPH employees, the doctors and midwives hold public roles with inherent responsibilities and accountability. Women in the community FGDs were clear, there were kind, sympathetic staff and there were cruel staff. Our findings concur with Jewkes and Penn-Kekana [59] that while blaming health workers as a group is not helpful, individual staff need to be held accountable for deliberate abuse or neglect. While the need to enforce standards appears an obvious statement, in the current cultural and political context of Afghanistan this is challenging to accomplish. The establishment of the Afghanistan Nurses and Midwives Council is clear progress towards professional regulation [60]. Although challenges to the status quo will not be easy, this is an important milestone for women’s health in Afghanistan.

### More nuanced

The doctors, midwives and care assistants in our research defied simple categorisation. Most staff members were simply endeavouring to survive in a tough working environment where the lack of a shift system inevitably resulted in staff exhaustion, poor performance and the constant risk of mistakes leading to censure by management. An increasing number of studies have also examined the perspectives of healthcare providers and documented similar struggles between the ideal and the limitations of staff energy, time, agency and the systems within which they work [49, 57, 61].

The picture that emerged of healthcare providers in our study was of a complicated and diverse group. It was not possible to typify villains or victims by their uniforms, as there were examples of both amongst the doctors, midwives and care assistants. Several studies have reported that doctors dominate midwives and restrict their scope of practice [58, 62, 63]. Our findings were more unusual, however, revealing that some senior midwives also prevented midwives from using particular clinical skills, and threatened resident doctors. There were extremes as some staff gained significant benefits, bullying and threatening other staff members as well as women in labour; at the other extreme were vulnerable staff members, with little agency due to a lack of connections, poor health, or family poverty, who worried about losing their jobs. In the middle were many midwives, doctors and care assistants, who at times suffered as victims but concurrently acted as villains towards colleagues or women in their care. Staff with chronic illnesses who struggled to do their work, for example, could also be stealing medicines and supplies sent to women in childbirth by their relatives. Rivkin-Fish [64] encountered a similar dichotomy in Post-Soviet Russia between the suffering of women in childbirth at the hands of healthcare providers and the frustrations and struggles of the same healthcare providers that contributed to their treatment of women. While recounting the distressing attitudes and behaviours of the staff she also portrayed their humanity and kindness to her.

### The inadequacy of binary thinking

This study revealed the inadequacy of binary thinking and problem solving in issues of human behaviour and interactions. If the definition of the problem is too narrow or rigid then the danger is of being locked into one perspective and way of thinking, new ideas will be difficult to generate which will inevitably lead to using the same kind of solutions. [65]. The frequent use of training is an example. This solution suggests that those designing and providing these interventions assume that healthcare providers are ignorant and that simply educating them will lead to a change. Even where training may be helpful, simply running courses is not sufficient, it is vital to ensure that different participants attend on each occasion and that their selection is based on need not connections to the hierarchy [52]. Similarly, Sadler and colleagues ( [66], p51) encouraged moving away from another binary mind-set - one that frames women as “victims” and health professionals as “victimisers”(villains). This limited focus, they argued, needs to be replaced by a broader analysis of the cultural, social and institutional factors that affect both women and health professionals and can lead to obstetric violence.

Cultural and structural factors influenced healthcare providers and impeded quality maternity care in Namibian hospitals and resulted in negative interactions with women in labour [50]. Structural issues such as the mismatch between staffing levels and workload in our study also contributed to staff exhaustion and poor interactions between staff and women in childbirth. This was illustrated by a doctor who tried to be kind but admitted to shouting at the tenth or fifteenth ‘patient’ due to sheer overload. From our findings we suggest that it is not sufficient to solely blame or conversely to excuse healthcare providers, rather it is necessary to compare the responsibilities of individual staff members alongside their actual ability and agency as well as their behaviour. Healthcare providers must be viewed as one element in the wider picture that includes all stakeholders, particularly those with overall responsibility to care for the health of their citizens. It is important to determine if the working environment enables doctors, midwives and care assistants to provide quality care or asks the impossible from them. Management should enforce the standards, reward those who are working well, refuse to tolerate poor care and ensure that systems, staffing levels and vital supplies make quality care possible. For outsiders, there is the need to examine their own assumptions regarding staff behaviours and the root causes of sub-optimal care, as well as working towards a more reflective practice.

Our study highlights the need for an even-handed approach to Afghan healthcare providers, to acknowledge their capacity for kindness and for cruelty, professionalism and mistreatment, altruism and selfishness. Both doctors and midwives stressed the need to acknowledge and encourage those who were working well and to ‘punish’ those who were deliberately negligent or abusive. Unless there were consequences they said, nothing would change. Unless there were penalties for professional negligence or malpractice and encouragement for those who were working well staff would become demotivated. Similarly, Entezar ( [67], p30) claims that “*force rather than persuasion*” is the language that Afghans understand and respect.

The findings from this research concur with Kruk and colleagues [9] that fixes at the micro-level alone (i.e. healthcare provider, clinic) are not enough to achieve high quality care; there is also the need for a system-wide analysis and improvement strategies. At the micro level of staff performance, however, Kruk and colleagues’ recommendations of competence-based clinical education, training in ethics and respectful care [9] have thus far not produced the desired outcomes in Afghanistan.

#### Strengths and limitations

A limitation of this research is that only one hospital was studied. Women in the FGDs, however, had given birth in various Kabul maternity hospitals and there were no detectable differences. Background interviewees also confirmed that institutional cultures are similar across Afghan public maternity hospitals. We would therefore suggest that the findings are transferable to other Afghan maternity hospitals.

Although findings from this research are unique to Afghanistan, the drivers of suboptimal care and mistreatment of women in other LMICs are likely to involve similar complicated groups of healthcare providers and imperfect health systems [12, 49, 50]. It is probable that aspects of the context and findings will resonate with other settings and thus be transferable beyond Afghanistan.

As an outsider, a non-Afghan working through an interpreter, RA (who had basic understanding of the language), could have unintentionally misunderstood and therefore misrepresented the health system and staff. With this in mind, throughout data collection and analysis the findings were checked with Afghan and foreign colleagues. Interviews were also double checked by an Afghan researcher to ensure accuracy and completeness.

## Conclusions

The research demonstrated that the drivers of Afghan healthcare provider performance and behaviours are highly complex, interrelated and multifaceted. Individual staff had the capacity for kindness and altruism but also for neglect and cruelty. Initiatives to improve standards of care in maternity services, therefore, need to move beyond two polar positions that view healthcare providers as either villains who must be re-trained, educated and disciplined, or victims who must be excused and helped. Binary thinking that frames healthcare providers in modes of villain or victim does a disservice to them as it denies their humanity, the complexity of their lives, struggles and the difficult daily choices that they are forced to make. It also disregards weaknesses in management and the wider health system. Most important, it fails women in childbirth who put their lives into the hands of health services because binary thinking is unlikely to produce the creative broad solutions that will drive transformation.

This study suggests that providing high quality respectful care for Afghan women in childbirth requires initiatives that encompass the gamut of individual health worker behaviour. This will require strong enforcement of standards and consequences for deliberate neglect or extortion, as well as support and acknowledgement for staff members who are working well. Most importantly, hospital managers and the MoPH need to provide all aspects of an enabling environment without which optimum care cannot take place.

Health systems are complex and function at multiple interconnected levels. Hence approaches are required to identify and address all facets of poor quality care and mistreatment, those related to individual professional accountability as well as those belonging to the domain and accountability of management, government and society.

## Data Availability

The datasets generated during this study are not publicly available because study participants did not consent to public availability. It would also be difficult to ensure the anonymity of participants in a close interconnected community.

## Abbreviations

FGD: Focus group discussion
LMICs: Low, and middle-income countries
MoPH: Ministry of Public Health
